# Alteration of gut microbiota in wild-borne long-tailed macaques after 1-year being housed in hygienic captivity

**DOI:** 10.1038/s41598-023-33163-6

**Published:** 2023-04-10

**Authors:** Vorthon Sawaswong, Prangwalai Chanchaem, Taratorn Kemthong, Saradee Warit, Angkana Chaiprasert, Suchinda Malaivijitnond, Sunchai Payungporn

**Affiliations:** 1 Program in Bioinformatics and Computational Biology, Graduate School, Chulalongkorn University, Bangkok, 10330 Thailand; 2 Center of Excellence in Systems Microbiology, Department of Biochemistry, Faculty of Medicine, Chulalongkorn University, 1873 Rama IV Road, Patumwan, Bangkok, 10330 Thailand; 3 Nucleic Acid Section, Laboratory of Metabolism, Center for Cancer Research, National Cancer Institute, National Institutes of Health, Bethesda, MD 20892 USA; 4 National Primate Research Center of Thailand, Chulalongkorn University, Saraburi, 18110 Thailand; 5 Industrial Tuberculosis Team, Industrial Medical Molecular Biotechnology Research Group, National Center for Genetic Engineering and Biotechnology, National Science and Technology Development Agency, Pathum Thani, 12120 Thailand; 6 Office for Research and Development, Faculty of Medicine Siriraj Hospital, Mahidol University, Bangkok, 10700 Thailand; 7 Department of Biology, Faculty of Science, Chulalongkorn University, Bangkok, 10330 Thailand

**Keywords:** Zoology, Microbial communities

## Abstract

The wild-born long-tailed macaques (*Macaca fascicularis*) were recently recruited and used as breeders for the National Primate Research Center of Thailand, Chulalongkorn University (NPRCT-CU), and changes in their in-depth gut microbiota profiles were investigated. The Oxford Nanopore Technology (ONT) was used to explore full-length 16S rDNA sequences of gut microbiota in animals once captured in their natural habitat and 1-year following translocation and housing in a hygienic environment at NPRCT-CU. Our findings show that the gut microbiota of macaques after 1 year of hygienic housing and programmed diets feeding was altered and reshaped. The prevalent gut bacteria such as *Prevotella copri* and *Faecalibacterium prausnitzii* were enriched after translocation, causing the lower alpha diversity. The correlation analysis revealed that *Prevotella copri*, *Phascolarctobacterium succinatutens*, and *Prevotella stercorea*, showed a positive correlation with each other. Significantly enriched pathways in the macaques after translocation included biosynthesis of essential amino acids, fatty acids, polyamine and butanoate. The effects of microbiota change could help macaques to harvest the energy from programmed diets and adapt their gut metabolism. The novel probiotics and microbiota engineering approach could be further developed based on the current findings and should be helpful for captive animal health care management.

## Introduction

The long-tailed macaque (*Macaca fascicularis*) is Thailand's most common macaque species and is widely distributed in Southeast Asian countries^1^. This macaque is also the non-human primate (NHPs) species widely used in biomedical research, including drug and vaccine trials^2,3^. Recently, captive macaques reared in research facilities worldwide have faced problems of diarrhea^4,5^, potentially caused by gut microbiota dysbiosis^6^. The gut microbiota has been recently shown to be influenced by many factors, including habitat, diet, disease, and antibiotic usage^7^. Generally, wild animals usually harbor a variety of microbes due to environmental exposure^8^. The gut bacterial microbiome in wild-living animals plays beneficial roles in gut immune modulation and pathogen resistance^9,10^. Therefore, rearing an animal within the hygienic conditions of captivity in a research facility may lead to gut microbiota alteration and the loss of the protective roles of these bacteria. Although using probiotic supplements derived from healthy animals to treat diarrhea in macaques has been tried, diarrhea still persisted^4^. Accordingly, the study of bacterial microbiota would be critically helpful in understanding their role and could help to identify the basic information necessary for the development of appropriate therapeutics for macaques with diarrhea in primate centers.

Previously, our investigators compared the gut microbiome (including bacteria, fungi, and viruses) between healthy wild macaque populations and the captive macaque colony which was the 1-year translocated wild-borne population in the National Primate Research center of Thailand—Chulalongkorn University (NPRCT-CU)^11–13^. These studies suggested that housing conditions could be responsible for a significant influence on the macaque’s microbiota. However, the previous study compared the microbiome of macaques from two different origins/genotypes, which may not fully reflect the shift of microbiota caused by different rearing conditions. In addition, the bacterial classification in the previous study was only pursued to the genus level due to the limitation of short-read 16S sequencing based on the Illumina platform^14^.

The present study aimed to utilize the full-length 16S (V1–V9) sequencing based on Oxford Nanopore Technologies to investigate the microbiome alteration in wild-borne macaques after they were translocated from the natural habitat and housed in the hygienic environment at the primate research center for 1 year.

## Results

### Gut microbiota of wild-borne macaques based on the nanopore platform

**Figure 1:**
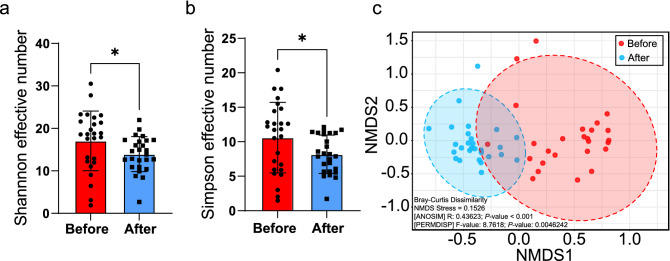
Comparison of alpha and beta diversity of gut microbiota in long-tailed macaques between before and after housing in hygienic captivity. The alpha diversity of gut microbiota from wild-macaques before and after translocation for 1 year were evaluated using (**a**) Shannon effective number and (**b**) Simpson effective number and statistically tested by *t*-test (*P* < 0.05). (**c**) The beta diversity based on Bray–Curtis dissimilarity is presented in a Non-metric Multidimensional Scaling (NMDS) plot showing the microbiota dissimilarity between different timepoints. The Analysis of similarities (ANOSIM) was used to statistically test the similarity of microbiota between time points with *P* < 0.05 while Permutational Multivariate Analysis of Dispersion (PERMDIS) statistically indicates the difference of community dispersion between time points (*P* < 0.05).

The alpha diversity of bacterial genera was evaluated based on the Shannon and Simpson effective numbers as presented in Fig. 1a,b. The Shannon effective number indicated that the diversity of bacterial genera in wild-borne macaques (Before; 17.07 ± 7.021%) was significantly higher (*P* = 1.78 × 10^−2^) than the 1-year captive housed macaques (After; 13.95 ± 4.143%). Similarly, Simpson's effective number in the Before set (10.61 ± 5.119) was significantly higher than (*P* = 3.37 × 10^−2^) in the After set (8.184 ± 2.781).

The NMDS plot based on Bray–Curtis dissimilarity showed that the gut microbiota component of macaques in their natural habitat differed from those in the macaques reared in the primate center for 1 year (Fig. 1c). The ANOSIM test exhibited a significant difference in microbiota between Before and After (R = 0.4362, *P* = 1.00 × 10^−4^. The difference in microbiota between groups was also consistently significant (ANOSIM, *P* = 1.00 × 10^−3^) in the generalized Unifrac analysis shown in Supplementary Fig. S1. Moreover, the dispersion of the microbiota component, as tested by Permutational Multivariate Analysis of Dispersion (PERMDIS), was also significantly different (F-value = 8.7618, *P* = 4.62 × 10^−3^). The dispersion of naturally living wild-borne macaques (Before) was higher than that in hygienic-housed macaques (After), indicating the larger variation of the gut bacterial component in the macaques living in their natural habitat.

### Comparison of the relative abundance of bacterial phyla and genera in wild-borne macaques before and after being housed in hygienic captivity

**Figure 2:**
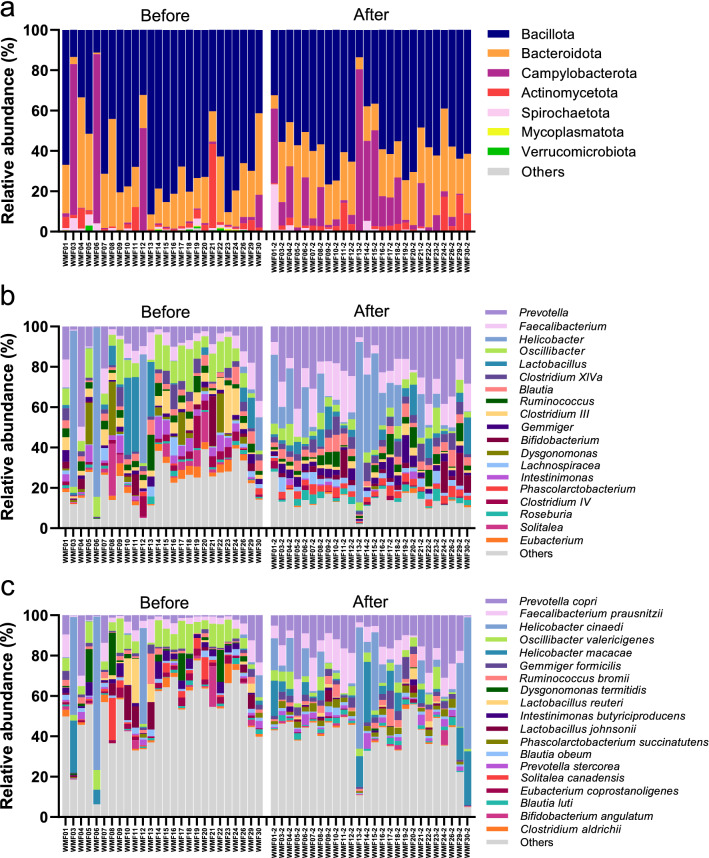
The relative abundance of dominant gut bacteria in wild-originated macaque before and after 1-year translocation into captivity. The bar plots showed the relative abundance (%) of gut microbiota based on Nanopore full-length 16S sequencing in wild macaques before and after translocation for 1 year. The abundant taxa from 3 taxonomic ranks, including (**a**) phylum, (**b**) genus and (**c**) species showed the alteration of microbiota after the macaques were translocated into the primate center for 1 year.

The relative abundances of gut microbiota are presented in Fig. 2a. The major bacterial phyla identified in macaques were similar between both living environments, Before and After captivity. These four major phyla include Bacillota, Bacteroidota, Campylobacterota, and Actinomycetota. The two most abundant phyla, Bacillota and Bacteroidota, contributed over 70% of total bacterial abundance. The phylum Bacillota in Before (62.75 ± 22.40%) showed a marginally greater relative abundance than in After (55.18 ± 14.35%; tested by paired *t*-test, *P* = 1.74 × 10^−1^). The relative abundance of the phylum Bacteroidota was not different between these two habitat types (Before: 21.99 ± 13.4% and After: 24.24 ± 9.14%). The remaining minor bacterial phyla, such as Spirochaetota, Mycoplasmatota, and Verrucomicrobiota, could be detected in some samples with low abundance (lower than 5% relative abundance). Interestingly, the phylum Mycoplasmatota abundance in naturally living wild-borne macaques (Before: 0.15 ± 0.23%) was significantly higher (*P* = 4.92 × 10^−2^) than that in the hygienic socially housed macaques (After: 0.05 ± 0.10%). Moreover, phylum Verrucomicrobia was also significantly enriched (*P* = 4.04 × 10^−3^) in the wild-borne macaques (0.38 ± 0.61%) compared to the 1-year captive housed macaques (0.003 ± 0.02%).

The relative abundance of bacterial genera shown in Fig. 2b indicated that the top abundant genera were altered. The most abundant genus was *Prevotella* which was significantly enriched (*P* = 1.94 × 10^−3^) in the After set (21.40 ± 10.00%) compared to the Before set (10.00 ± 11.36%). *Helicobacter* was the second most abundant genus (Before: 9.20 ± 23.57%, and After: 19.77 ± 24.54%). The third dominant genus was *Faecalibacterium* which also contributed the greater relative abundance in the After set (10.23 ± 6.02%) than in the Before set (5.56 ± 4.08%). *Lactobacillus*, *Clostridium XlV*, *Blautia*, and *Ruminococcus* genera (3–5% each) were identified in both habitat types. Two *Clostridium* genera, including *Clostridium III* and *Clostridium IV*, exhibited significantly higher abundance in the Before set (*P* = 1.49 × 10^−3^ and* P* = 4.98 × 10^−3^, respectively). Some bacterial genera such as *Gemmiger*, *Phascolarctobacterium*, and *Roseburia* were significantly enriched in the After set (*P* = 1.19 × 10^−2^, *P* = 1.78 × 10^−8^, and *P* = 4.30 × 10^−2^, respectively).

### Differential abundance of bacterial species in gut microbiota before and after being housed in hygienic captivity

**Figure 3:**
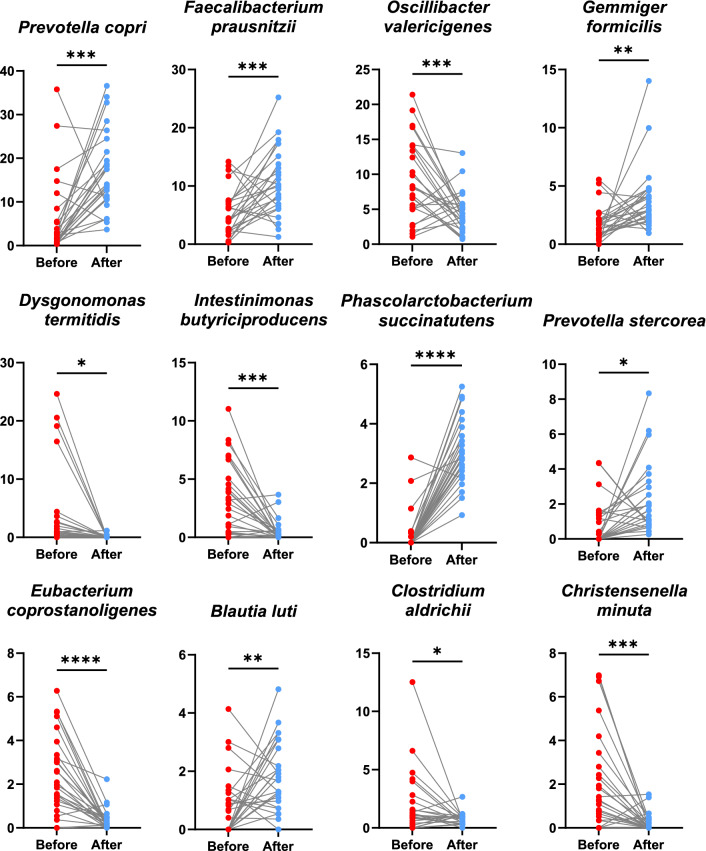
Longitudinal analysis of relative abundance of top abundant bacteria in the macaque before and after 1-year translocation into hygienic housing. The scatter dot plots show the significant alteration of top abundant bacteria (> 1% relative abundance) in the wild macaques ‘before’ (red) compared to ‘after’ (blue) 1-year translocation, as evaluated by dependent *t*-test (*P* < 0.05).

The overview of the top 20 bacterial species abundance was illustrated in Fig. 2c. The comparison of species abundance between the two different habitat types was primarily performed using Linear discriminant analysis Effect Size (LEfSe) with settings of *P* < 0.05 and LDA > 4.5, as shown in Supplementary Table S3 and Supplementary Fig. S2. To investigate the shifting of bacterial species in wild-borne long-tailed macaques during 1 year of housing in a hygienic environment, the most abundant significant bacteria from LEfSe were also longitudinally tested using a dependent *t*-test (*P* < 0.05), as shown in Fig. 3. The results showed that *Prevotella copri*, *Faecalibacterium prausnitzii*, *Phascolarctobacterium succinatutens*, *Gemmiger formicilis*, *Prevotella stercorea*, and *Blautia luti* were significantly increased after the macaques were reared in a hygienic research facility for 1 year. Nevertheless, several bacterial species, including *Oscillibacter valericigenes*,* Dysgonomonas termitidis*, *Intestinimonas butyriciproducens*, *Christensenella minuta*, *Eubacterium coprostanoligenes*, and *Clostridium aldrichii*, were significantly decreased after translocation and housing in hygienic captivity.

### The correlation network analysis of gut bacterial species in long-tailed macaques

**Figure 4:**
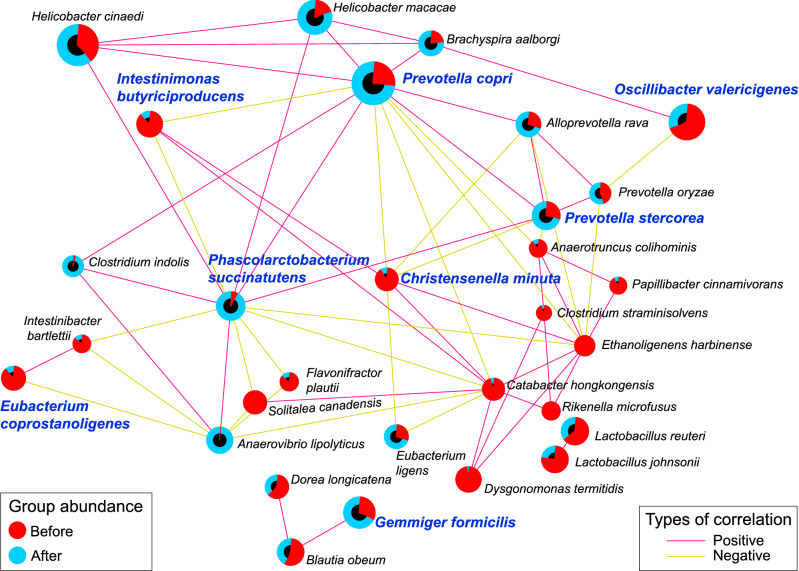
Correlation network analysis of bacterial species in the gut microbiota of wild-borne macaque before and after 1-year translocation. The correlation network constructed based on the SPARCC method implies the relationship of bacterial species in the gut microbiota of macaques (*r* > 0.6, *P* < 0.05). The edges colors represent the types of correlation: positive (pink) and negative (yellow) correlation. The pie charts were used as the nodes in the network, which indicate the relative abundance compared between the before (red) and after (blue) sets. The node sizes represent the overall relative abundance of each bacteria within the community. The bacterial names highlighted in blue text are the significantly changed species from the longitudinal differential abundance analysis.

The Strong, Prosperous, And Resilient Communities Challenge (SPARCC) analysis was performed to investigate the relationship between gut bacteria in long-tailed macaques. The significant correlations between bacterial species (*r* > 0.6, *P* < 0.05) were presented as the correlation network in Fig. 4. The significant species from differential abundance analysis, including *P*. *copri*, *P. succinatutens*, *G. formicilis*, *P. stercorea*, *O. valericigenes*,* I. butyriciproducens*, *C. minuta*, and *E. coprostanoligenes* were shown in the network. The species enriched in the after set, including *P. copri*, *P. succinatutens*, and *P. stercorea*, showed a positive correlation between each other and with other species, such as *Helicobacter cinaedi*, *Helicobacter macacae*, and *Brachyspira aalborgi*. Interestingly, *C. minuta* and *I. butyriciproducens* enriched in wild-living macaque showed a negative correlation to *P. copri*, *P. succinatutens*, and *P. stercorea* group. Moreover, *C. minuta* also positively correlated with *Catabacter hongkongensis* and *Ethanoligenens harbinense* located in the cluster of species enriched in the wild-living group.

### Functional inference analysis of gut microbiota in long-tailed macaques before and after translocation

**Figure 5:**
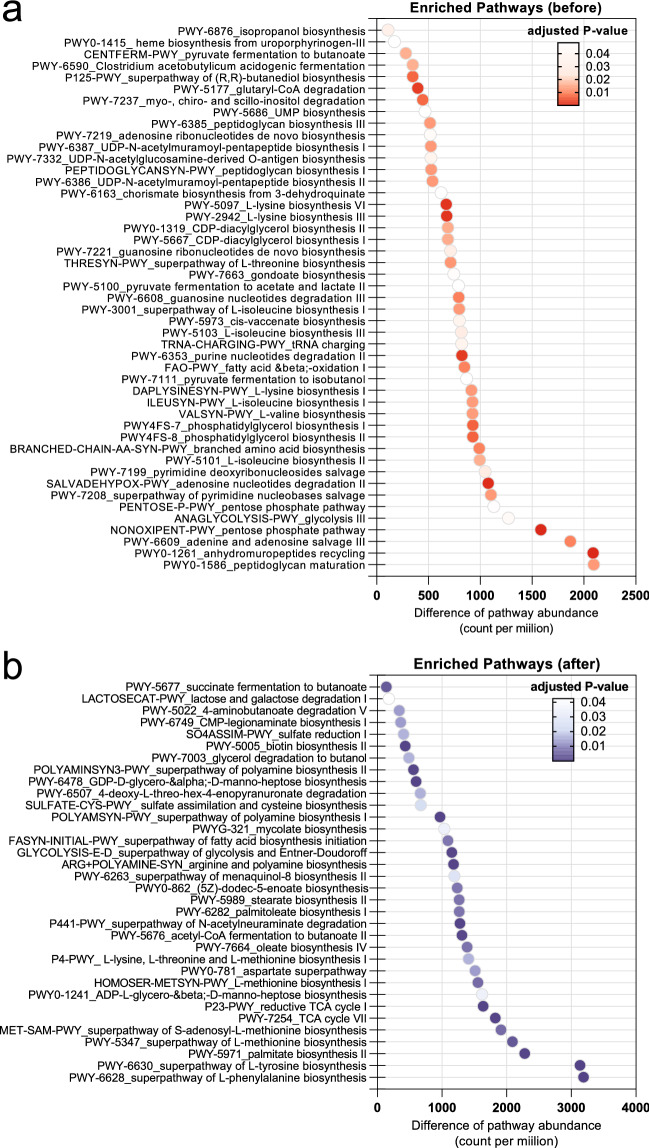
Functional inference of gut microbiota in the macaques living in wild habitat and macaques in a hygienic environment. The dot plots show the significantly enriched microbial pathways (multiple *t*-tests, two-stage step-up method of Benjamini Krieger and Yekutieli, adjusted *P* value < 0.05) based on PICRUSt2 analysis and MetaCyc pathway annotation in (**a**) wild-living macaques before translocation and (**b**) macaques living in hygienic captivity after translocation. Differences in average abundances were plotted on the X-axis, while the color levels of the dot represent the adjusted *P* value.

The significant MetaCyc pathways (multiple *t*-tests, two-stage step-up method of Benjamini Krieger and Yekutieli, adjusted *P* value < 0.05) based on functional inference by PICRUSt2 were shown in Fig. 5. The enriched pathways in the gut microbiota of macaques before translocation were shown in Fig. 5a. The pentose phosphate pathway (PENTOSE-P-PWY and NONOXIPENT-PWY) and the glycolysis pathway of anaerobes (ANAGLYCOLYSIS-PWY) were highly enriched in wild-living macaques (Before). In addition, FAO-PWY fatty acid and beta-oxidation pathway was also enriched in these macaques. Interestingly, several pathways related to the synthesis of essential amino acids, including lysine, threonine, isoleucine and valine (e.g. PWY-5097, PWY-2942, THRESYN-PWY, PWY-3001, PWY-5103, ILEUSYN-PWY and VALSYN-PWY) were significantly higher in the macaques living in natural habitat. The short-chain fatty acid production pathways include CENTFERM-PWY (pyruvate fermentation to butanoate), and PWY-5100 (pyruvate fermentation to acetate and lactate II), were significantly enriched in these macaques.

The significantly enriched pathways in the macaques after translocation were shown in Fig. 5b. Several top enriched pathways related to some essential amino acid synthesis were found, such as l-phenylalanine biosynthesis (PWY-6628), methionine synthesis (PWY-5347, MET-SAM-PWY, and HOMOSER-METSYN-PWY). Moreover, the pathways involving the fatty acid synthesis were observed, including palmitate biosynthesis (PWY-5971), oleate biosynthesis (PWY-7664), stearate biosynthesis II (PWY-5989), palmitoleate biosynthesis (PWY-6282), (5Z)-dodec-5-enoate biosynthesis (PWY0-862) and superpathway of fatty acid biosynthesis initiation (FASYN-INITIAL-PWY). The polyamine biosynthesis pathways, including POLYAMSYN-PWY, ARG + POLYAMINE-SYN and POLYAMINSYN3-PWY were significantly enriched in the macaques living in the hygienic environment (After). The pathways associated with butanoate synthesis, including PWY-5677 and PWY-5022 were also higher in these macaques.

## Discussion

This study incorporated the full-length 16S sequencing approach using the Oxford Nanopore Technology to study the alteration of gut microbiota in the wild-borne Thai long-tailed macaques living in the natural habitat (Before) and after being translocated to be socially housed in hygienic captivity (After) at the AAALAC International accredited facility. The full-length 16S sequencing workflow based on Oxford Nanopore Technology has the advantage of greater efficiency in species classification^15^. This strategy is also cost-effective^16^ due to the cheaper reusable flow cells, more flexible input sample requirement and a rapid turnaround time^17^. At present, we investigate the alteration of the gut microbiome in wild-borne macaques that were translocated and housed in a hygienic facility for 1 year. The beta diversity suggested that the gut microbiota of macaques had shifted from the original profile and had less variation. These results indicated that the effect of habitat and diet changes for 1 year could reshape the gut microbiota of long-tailed macaques. This is in line with the published study that found captive animals have different microbiota profiles compared to the same species living in its natural habitat^18,19^. It was previously known that aging is also one-factor influencing gut microbiota^20^. However, altering microbiota by aging usually depends on personal factors^21^. In addition, diets and extrinsic factors such as social contact and environment seem to have a larger effect rather than aging^21,22^. For the alpha diversity, both Shannon and Simpson's effective numbers decreased after translocation. The result suggested that the diet and environment changes may cause the macaques to acquire different bacterial species and numbers, altering the bacterial diversity. In addition, the hygienic housing might enrich some dominant bacteria, especially *P. copri*, *F. prausnitzii*, and *P. succinatutens*, causing a lower diversity of transition microbiota.

The *P. copri* is one of the most common gut bacteria found in humans, with ~ 40% prevalence^23^. This species is associated with high carbohydrate and low-fat consumption^24^. This result implies that the diets provided to macaques in the primate center might contain more carbohydrates than those in the natural habitat. There has been controversy about this bacteria's beneficial and adverse effects on the host's health. Generally, *P. copri* is known for its complex-polysaccharides metabolizer functions^25^. This species is the main succinate producer providing succinate for intestinal gluconeogenesis, which has been found to improve glucose homeostasis^26^. Nevertheless, some previous studies demonstrated that *P. copri is* likely associated with mucosal inflammation^27^, insulin resistance^28^, and susceptibility to arthritis^29^. Another study suggested that *P. copri* positively correlates with fat accumulation and altering serum metabolites in pigs fed with a formula diet^30^. Therefore, *P. copri* might be an important microbe in relation to energy harvest and utilization by gut microbiota. The enrichment of *P. stercorea* was also observed in the hygienic-housed macaques. This species was the second most abundant *Prevotella* species identified in the human gut, which cooperatively facilitates *P. copri* in metabolizing dietary fiber using ester modifications for carbohydrate degradation^25^.

The *F.* is an important abundant commensal bacteria found in the human colon with approximately 5% relative abundance^31^. It has been reported as the main butyrate producer, serving as the main energy source of colonocytes, and modulating the anti-inflammatory response^32^. It was suggested that the *F. prausnitzii* was related to the consumption of high-fiber diets, the fermentable substrate for short-chain fatty acid production^33^. Therefore, increasing *F. prausnitzii* in the hygienic housed macaques could enhance the beneficial functions of the macaque's gut. It could be a potential probiotic supplement for captive non-human primates with gut dysbiosis. The *P. succinatutens*, a succinate-utilizing bacterium, was previously isolated and identified from the healthy human gut^34^. Typically, this species cannot ferment other carbohydrates and fatty acids except succinate^34^. The closely related species, *Phascolarctobacterium faecium* also exclusively uses succinate produced by other bacteria (such as *Bacteroides* species) as the substrate for propionate production^35^. In the current study, we hypothesized that increasing *P. copri*, the main succinate producer in these macaques, may promote the colonization of *P. succinatutens.* Propionate is known to be an important short-chain fatty acid (SCFA) that could lower lipogenesis and carcinogenesis^34,35^. Accordingly, this bacterial species may be the potential key regulator controlling microbial-produced succinate and be an important propionate producer^34^. *G. formicilis* is also a carbohydrate fermenter that mainly produces formic and butyric acids, but its roles have not been extensively studied^36^. *Blautia* is a genus of beneficial Bacillota which has been proposed for their probiotic properties, contributing important roles in inter-species crosstalk, biotransformation and ameliorated inflammatory diseases^37^. *B. luti*, enriched in our translocated macaques, is one of the most abundant *Blautia* species detected in the human gut^38^. A previous study found that depletion of *B. luti* was associated with the obesity phenotype in children and metabolic inflammation, causing insulin resistance^39^.

In the present study, several dominant bacteria such as *O. valericigenes*,* D. termitidis*,* I. butyriciproducens*, *C. minuta*, *E. coprostanoligenes* and *C. aldrichii* were markedly depleted after animals were translocated from the natural habitat to a captive hygienic habitat*.* The *O. valericigenes*, a species first identified from the gut of invertebrates^40^, was enriched in the gut of healthy controls compared to people with Crohn's disease^41^. However, the functions of this bacteria are still not well explained. The *D. termitidis* is the commensal bacteria found in the gut of wood-feeding termites^42^. This species can mainly ferment soluble starch and xylan, the component of plant cell walls^42^. Thus, some wild-borne macaques may consume termites or plant debris containing this bacterium. However, this species is mostly absent in captive housed animals (After), suggesting that it could not colonize and survive in a macaque gut environment. The *C. aldrichii* is a cellulolytic bacterium that utilizes cellulose, xylan, and cellobiose for fermentation which produce several kinds of short-chain fatty acid such as acetate, propionate, butyrate, and lactate^43^. Thus, this bacterium may be the diet degrader facilitating the digestive system to extract energy from plant-rich diets. One of the butyrate-producing species, *I. butyriciproducens* was detected and enriched in several macaques living in the natural condition. This species could contribute to the beneficial roles of modulating gut homeostasis^44^. Although it was decreased, their functions could be compensated by the functions of *F. prausnitzii*, a closely related butyrate producer, increasing in the translocated macaques. Another depleted species, *C. minuta*, exhibited anti-inflammatory effects by producing acetate and modulating the level of butyrate, inhibiting the NF-κB signaling pathway and suppressing the level of IL-8 cytokine^45^. Recently, this species has been proposed as a promising probiotic candidate for therapeutics of Crohn's disease (CD)^46^. Moreover, it was also a potential biotherapy to diminish obesity and metabolic disorders^47^. Accordingly, this species would also be a potential candidate for probiotics supplementation for modulating gut homeostasis against diarrhea problems in macaques reared in a primate center. Based on the correlation network analysis, *C. minuta* and *I*. *butyriciproducens* exhibited a negative correlation with the species enriched after translocation, including *P. copri*, which was reported to associate with mucosal inflammation^27^. This is convincible that *C. minuta* and *I*. *butyriciproducens* would be promising probiotic candidates. Previously, *Lactobacillus casei* isolated from healthy macaques was used as probiotic treatment for diarrheal macaques, but diarrhea persisted^4^. It was possible that the *Lactobacillus* species is not the key modulator of microbiota in long-tailed macaques. Unlike *C. minuta* and *I*. *butyriciproducens*, our correlation network showed that *Lactobacillus* spp. was not associated with the abundant core microbiota. Therefore, it may not be surprising that *Lactobacillus* was ineffective in these macaques. Another interesting species depleted after translocation is *E. coprostanoligenes.* It was known as a cholesterol-reducing bacteria^48,49^. Accordingly, it may be the probiotic candidate for cholesterol reduction in these macaques. The current findings may help understand the complex relationship of gut microbiota in macaques which will be the basis for developing therapeutics or dietary supplements for reared macaques. For example, these candidate probiotics might be administered by mixing with the chow diet fed to the macaques to engineer a healthy microbiota environment in macaques reared in primate centers. However, further study is still needed to validate the efficiency and effects of these microbes.

In this study, we identified *Helicobacter* species, including *H. cinaedi* and *H. macacae* enriched in hygienic housed macaques, based on LEfSe analysis. Both species have been detected in the captive rhesus macaques (*Macaca mulatta*), the close relative species of long-tailed macaques^50,51^. Typically, *H. cinaedi* has also been isolated from a wide range of animal species and was considered an opportunistic pathogen, especially in immunocompromised patients^52^. This species was previously found in the colon, liver, and mesenteric lymph nodes in rhesus macaques with chronic diarrhea and hepatitis^53^. Similarly, *H. macacae* was an enterohepatic pathogen isolated in the rhesus macaques with idiopathic diarrhea and intestinal adenocarcinoma^54^. Therefore, these species might be the potential enzootics and zoonotic pathogens that need to be monitored and eliminated for animal health care. However, our cohort was physically healthy, suggesting that these species could be opportunistic pathogens causing disease in an immunocompromised host. In addition, it further implied that the gut microbiota of our macaques could still efficiently modulate the mucosal immune and gut stability to suppress the pathogenesis of these potential pathogens.

The functional inference performed in this study suggested in potential roles of different microbiota profiles before and after translocation. We identified the enriched biosynthesis pathways of essential amino acids, including lysine, threonine, isoleucine and valine, in the macaques living in wild habitats. It was known that gut microbiota facilitates the de novo synthesis of essential amino acids, affecting the host's amino acid homeostasis^33^. The microbial-derived lysine and threonine were found to be absorbed and circulated in the plasma of healthy individuals^55^ suggesting that the microbiota might be the source of essential amino acids for wild macaques. Threonine not only be the precursor for host metabolism, but it could also modulate the intestinal immune functions as described previously^56^. The microbial-derived branched-chain amino acids (BCAAs) such as isoleucine and valine were known to be correlated with the obesity and insulin resistance phenotypes^57^. However, the enrichment of microbial BCAAs pathway may not cause adverse effects on macaques, but it might be the source of essential amino acids, which may be lacking in natural food sources. Our finding suggested that the gut microbiota of wild-living macaques probably generate short-chain fatty acids, including butyrate and acetate, mainly via pyruvate fermentation (Fig. 5a). In contrast, the macaques living in hygienic conditions enriched the microbial pathway producing butyrate via succinate fermentation and 4-aminobutanoic acid degradation (Fig. 5b). This implied that the SCFAs production might be altered due to microbiota change after translocation. The succinate producer, *P. copri* was increased, implying that this may cause the microbiota and metabolic shift to maintain the gut homeostasis in these macaques. It was found that the microbiota of captive-housed macaques (after) enrich some amino acid biosynthesis pathways (including methionine, tyrosine and phenylalanine synthesis). The effect of these amino acids produced by gut bacteria might be two-sided and controversial. For example, methionine has been reported to improve mucosal villus architecture and reduce the risk of colon cancer^58^. However, other studies suggested that methionine restriction could improve gut barrier functions^59^ and longevity^60^. After translocation, we found that the microbiota of macaques enriched with the pathways for long-chain fatty acid synthesis, e.g. palmitoleate, oleate, stearate and palmitate. It was found that some saturated long-chain fatty acids, such as stearic acid derived from gut bacteria, can modulate colonic motility in rats^61^. In contrast, palmitate was reported to disrupt gut epithelium integrity and elevate the production of inflammatory cytokines^62^. Therefore, it would be interesting to investigate further the role of fatty-acid production in these macaques, which might reflect the animal health status. Lastly, the polyamines (PA) biosynthesis is also abundant in macaques living in a hygienic environment. It has been reported that gut microbiota-derived bacteria could suppress chronic inflammation and protect the intestinal barrier^63,64^. This suggested that the changed microbiota may regulate gut stability via polyamine metabolism. Based on the functional analysis, it could be summarized that altered microbiota after 1-year translocation may adapt the metabolic function in response to dietary and environmental changes. The altered functions seem to have no obvious adverse effects on these macaques. It was suggested that microbiota could maintain gut homeostasis and reserve the essential metabolic functions in these macaques. For example, the short-chain fatty acid productions were also maintained, but the mechanisms were changed. However, it is still interesting to follow up on the long-term effects of captivity on this macaque in the future.

In summary, our current findings represent useful information presenting the alteration of gut microbiota as affected by the translocation of wild-borne macaques from the natural habitat into hygienic captivity. The full-length 16S sequencing applied in this study could be further used to monitor the macaque microbiota and other captive animals with species-level resolution. Finally, the information on baseline microbiota in wild-borne macaques can benefit the further development of gut microbiota reconstitution approaches to modulate gut stability and mucosal immunity in the captive macaque colonies. One of these approaches may be the development of probiotics based on the present findings, and which could alleviate the endemic diarrhea of captive macaques reared in primate centers worldwide.

## Methods

### Animal experiment statement

All procedures were carried out under the relevant guidelines and regulations. This study's procedure related to macaques was performed and reported following the ARRIVE guidelines (https://arriveguidelines.org/arrive-guidelines). The animal use protocol for a sample collection from macaques was reviewed and approved by the NPRCT-CU animal care and use committee (Protocol Review No. 1775005).

### Animal cohorts

The cohort of wild-originated cynomolgus macaques (n = 26 from Wat Tham Praporthisat, Saraburi, Thailand (GPS: 14°34′N, 101°08′E) was recruited for longitudinal analysis of gut microbiota. The capture of macaques was approved by the Department of National Parks, Wildlife and Plant Conservation; permission no. 0909.702/1431 (25 Jan 2016) and 0909.302/5369 (25 Mar 2014). The age of macaques ranged from 1.5 to 18 years. The details of macaque characteristics were described in a previous study^14^ and Supplementary Table S1. All macaques recruited in this study were healthy and had no background of diarrhea. For captivity conditions, after the translocated macaques were quarantined for specific pathogen screening, they were moved to the semi-open stainless steel gang cages (4 m × 4 m × 3 m; W × L × H) and socially housed in a controlled hygienic environment. The cage facilities were supplied with hyperchlorinated water (1 ppm) through automatic lixit and cleaned with high pressure water two times per day. Flooring was coated with polyurethane. The macaques were fed under a food provisioning program with the standard monkey chow (Perfect Companion Group Co., Ltd, Thailand) in the morning at 9–10 AM, and fresh fruits (such as melons, bananas, dragon fruits, watermelons, guava and oranges) in the afternoon at 2–3 PM. The caloric intake was calculated based on the Committee on Animal Nutrition Ad Hoc Committee on Nonhuman Primate Nutrition Board on Agriculture and Natural Resources Division on Earth and Life Studies (2003). The NPRCT-CU is an AAALAC International accredited facility (AAALAC International Accredited: 1752).

### Specimen collection

The fecal swab samples were collected from 26 wild-borne long-tailed macaques once they were captured in their natural habitat (Before) and again 1 year after being housed at the NPRCT-CU (After). The macaques were anesthetized by an experienced veterinarian using a mixture of dexmedetomidine hydrochloride (Zoetis, USA) (0.03–0.05 mg/kg) and Zoletil (Virbac, New Zealand) (3–5 mg/kg). The fecal swabbing was done directly at the rectum of the anesthetized animal using polyester-tipped swabs (Puritan, USA). The fecal swab was then immediately preserved in the freshly prepared viral transport media (VTM) and transported to the laboratory within 12 h. The VTM mixture was prepared as described in a previous study^14^. The fecal suspension was stored at − 80 °C until being used for DNA extraction.

### The full-length 16S library preparation and sequencing based on Oxford Nanopore Technology

The procedure for full-length 16S sequencing was as described in a previous study^65^. Briefly, total DNA extraction was performed using a ZymoBIOMICS DNA Miniprep Kit (Zymo Research, USA) according to the manufacturer's protocol. The full-length 16S rDNA (V1-V9 regions) of bacteria was PCR amplified using the developed in-house methods based on the primers from a previous publication^66^. The primers contain the sequences of specific target primers (underlined) and 5′ nanopore adaptor tails as follows: 16S_27F 5′-TTTCTGTTGGTGCTGATATTGCAGRGTTYGATYMTGGCTCAG-3′ and 16S_1492R 5′-ACTTGCCTGTCGCTCTATCTTCCGGYTACCTTGTTACGACTT-3′. The 20 µL PCR reaction comprised 0.25 µM of each forward and reverse primer, 0.2 µM of dNTPs, 0.4 U of Phusion™ Plus DNA Polymerase (Thermo Fisher Scientific, USA) and 10 ng of DNA template. The PCR was performed using the following thermal profile: 98 °C for 30 s, 25 cycles of 98 °C for 10 s, 60 °C for 10 s, 72 °C for 45 s, and a final extension at 72 °C for 5 min. Then, the DNA library from each sample was incorporated with the multiplexing Nanopore barcodes using the PCR Barcoding Expansion 1–96 (EXP-PBC096) kit (Oxford Nanopore Technologies, UK). The PCR barcode reaction was the same as the 1st step PCR, but with the barcode primers being used. The barcoding step was performed based on the 5-cycle PCR using the thermal profile of 98 °C for 30 s, 5 cycles of 98 °C for 10 s, 60 °C for 10 s, 72 °C for 60 s, and a final extension at 72 °C for 5 min. The barcode-tagged DNA was then purified using the QIAquick PCR Purification Kit (Qiagen, Germany) and quantified by Quant-iT™ dsDNA HS Assay Kits (Invitrogen, USA). The DNA was pooled equimolarly to get a total of 5 µg DNA pool. The pooled DNA was then purified using 0.5 × Agencourt AMPure XP beads (Beckman Coulter, USA) and eluted with 50 µL of elution buffer. The 47 µL containing 1 µg of DNA library was used for adaptor ligation using the Ligation Sequencing Kit (SQK-LSK112, Oxford Nanopore Technologies, UK) following the standard protocol. The final adaptor-ligated DNA library was loaded and sequenced on the R10.4 (Q20+) flow cell using the MinION Mk1C sequencer (Oxford Nanopore Technologies, UK). The sequencing read statistics was described in the Supplementary Table S2.

### Bioinformatic analysis of full-length 16S sequence data from nanopore sequencing

The raw FAST5 data of full-length 16S sequences from Nanopore sequencing was basecalled using a super-accuracy (SUP) mode of guppy basecaller v6.1.2 (Oxford Nanopore Technologies, UK) on a GPU-equipped workstation. The overview of FASTQ sequence quality was checked using the MinIONQC^67^. The demultiplexing and adaptor trimming was carried out using porechop v0.2.4 (https://github.com/rrwick/Porechop). The demultiplexed filtered reads were subsequently processed by NanoCLUST^68^ run on the Nextflow pipeline manager to perform the read clustering, polishing, and taxonomic identification. The minimum cluster size was set to 20, while the database for bacterial classification was the reference 16S sequences of known bacterial species retrieved from the RDP database (version 11.5). The taxonomic data and abundance information from NanoCLUST were collected and imported into the QIIME2 analysis pipeline (QIIME2 v2021.2)^69^ to collapse the taxa table using the QIIME2 implemented plugin. The rarefaction analysis was carried out using QIIME2 implemented plugin (the result was shown in Supplementary Fig. S3). The data were also analyzed and visualized using MicrobiomeAnalyst^70^. The correlation network analysis was performed based on the Strong, Prosperous, And Resilient Communities Challenge (SPARCC) method^71^ using the threshold of correlation coefficient (*r*) = 0.6 and *P* < 0.05.

### Comparison of bacterial microbiota in the gut of macaques before and after translocation based on 16S full-length sequencing

The longitudinal analysis of gut microbiota in the macaques before and after translocation was carried out based on 26 paired samples. The alpha diversity was determined based on Shannon and Simpson's effective numbers, which were statistically compared by the Kruskal–Wallis test (*P* < 0.05). The beta diversity was examined based on the Bray–Curtis dissimilarity and generalized Unifac distance using QIIME2 implemented plugin. The dissimilarity matrices were used for constructing Non-metric Multidimensional Scaling (NMDS) plot using the metaMDS function in Vegan R package^72^ and ggPlot2 package. The Analysis of similarities (ANOSIM) was used to test the statistical similarity of microbiota between time points (*P* < 0.05). The dispersion of the microbiota profile was evaluated by Permutational Multivariate Analysis of Dispersion (PERMDIS, *P* < 0.05). The differential bacterial enrichment was then analyzed using the linear discriminant analysis effect size (LEfSe)^73^ method run on the galaxy server (https://huttenhower.sph.harvard.edu/galaxy/) with the threshold of LDA score > 4.5 and *P* < 0.05. The pairwise analysis of top abundant bacteria was also carried out using a dependent (paired) *t*-test (*P* < 0.05, two-tailed) to investigate the longitudinal alteration of core microbiota. The PICRUSt2 was used for the functional prediction of microbiota based on the 16S sequences. The functional gene profiles from PICRUSt2 were subjected to infer the microbial pathway abundance based on MetaCyc pathway annotation^74^. The multiple *t*-tests using the two-stage step-up method of Benjamini Krieger and Yekutieli were performed (adjusted *P* value < 0.05) to investigate the significantly enriched pathways in each time point.

## Supplementary Information

Supplementary Information.

## Data Availability

The sequencing datasets used in this study are publicly available in the NCBI Sequence Read Archive (SRA), BioProject ID: PRJNA908263.
