# Supplementary material for: Alteration of gut microbiota in wild-borne long-tailed macaques after 1-year being housed in hygienic captivity
Source: Sci Rep. 2023 Apr 10;13:5842. doi: 10.1038/s41598-023-33163-6 (PMC10085984; doi:10.1038/s41598-023-33163-6)
Supplement: Supplementary file 1 — Supplementary Information. [file 41598_2023_33163_MOESM1_ESM.pdf]

Alteration of gut microbiota in wild-borne long-tailed macaques after one-year being housed in hygienic captivity

Vorthon Sawaswong[1,2], Prangwalai Chanchaem[2], Taratorn Kemthong[3], Saradee Warit[4], Angkana Chaiprasert[5], Suchinda Malaivijitnond[3,6], Sunchai Payungporn[2,*]

[1] Program in Bioinformatics and Computational Biology, Graduate School, Chulalongkorn University, Bangkok, 10330, Thailand.

[2] Center of Excellence in Systems Microbiology, Department of Biochemistry, Faculty of Medicine, Chulalongkorn University, Bangkok, 10330, Thailand.

[3] National Primate Research Center of Thailand, Chulalongkorn University, Saraburi, 18110, Thailand.

[4] Industrial Tuberculosis Team, Industrial Medical Molecular Biotechnology Research Group, National Center for Genetic Engineering and Biotechnology, National Science and Technology Development Agency, Pathum Thani, 12120, Thailand.

[5] Office for Research and Development, Faculty of Medicine Siriraj Hospital, Mahidol University, Bangkok, 10700, Thailand.

[6] Department of Biology, Faculty of Science, Chulalongkorn University, Bangkok, 10330, Thailand.

*Corresponding author:

Associate Professor Sunchai Payungporn, Ph.D.

Center of Excellence in Systems Microbiology,

Department of Biochemistry, Faculty of Medicine, Chulalongkorn University

1873 Rama IV Road, Patumwan, Bangkok 10330, Thailand

E-mail: sp.medbiochemcu@gmail.com Tel: +662-256-4482

Supplementary Table S1 The characteristics of the macaque population in this study

	n	Age (year) [a]	Body weight (kg) [a]
Female	9 (34.6%)	6.79±4.47	3.45±1.07
Male	17 (65.4%)	4.57±2.62	4.59±2.23
P value	0.12 [b]	0.17	0.22

[a] Age and Body weight were measured at the capture date and presented as mean ± SD with *P* value statistically tested by *t*-test. The missing data from 5 macaques were excluded from the analysis.

[b] The proportion of macaque number between male and female were tested based on the Chi-square test.

Supplementary Table S2 The sequence statistics of sequencing datasets full-length 16S based on the Nanopore platform

**Sequence statistics**	**Full-length 16S datasets based on Oxford Nanopore Technology**	
	**(Before translocation)**	**(After translocation)**
Number of samples	N=26	N=26
Raw reads	17,868±2,133	25,022±8,722
Filtered and classified reads	10,573±1,636	19,877±7,118
% Read classified	59.17±6.08	79.58±5.4

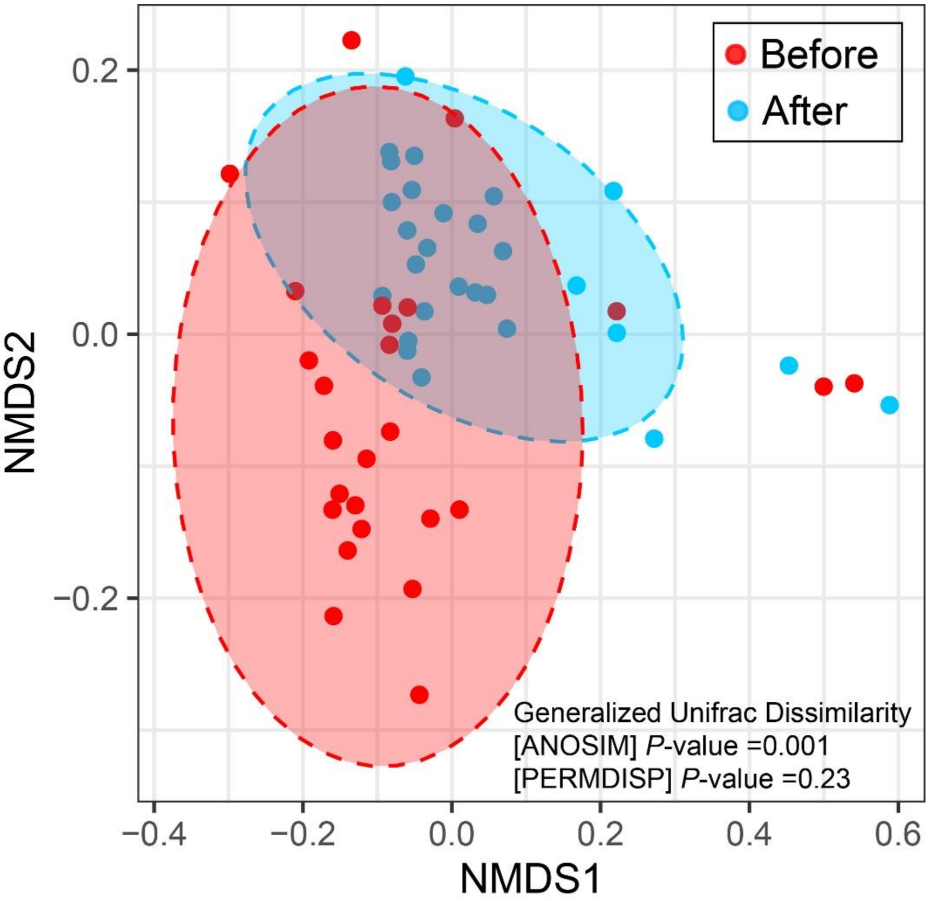

Supplementary Figure S1. The beta diversity based on generalized Unifrac dissimilarity is presented in a Non-metric Multidimensional Scaling (NMDS) plot showing the microbiota dissimilarity between different time points. The analysis of similarities (ANOSIM) was used to statistically test the similarity of microbiota between time points with $P<0.05$, while Permutational Multivariate Analysis of Dispersion (PERMDIS) statistically indicates the difference of community dispersion between time points ($P<0.05$)

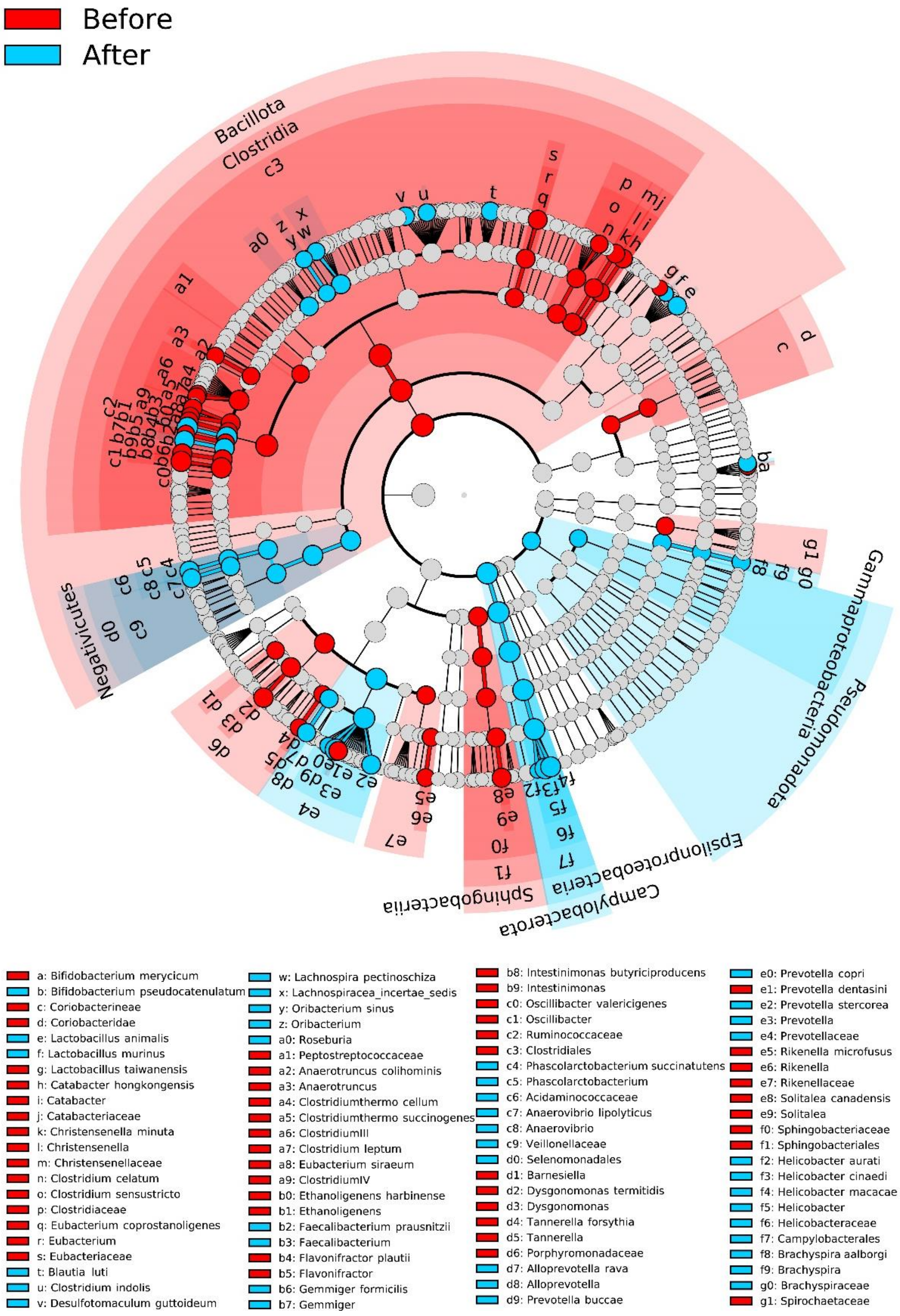

Supplementary Figure S2. Differential abundance analysis comparing bacterial taxa in wild macaque before and after translocation into the primate center for 1 year.

The cladogram illustrated the results from Linear discriminant analysis Effect Size (LEfSe) analysis showing the significantly differential taxa (LDA score > 3.5, P<0.05).

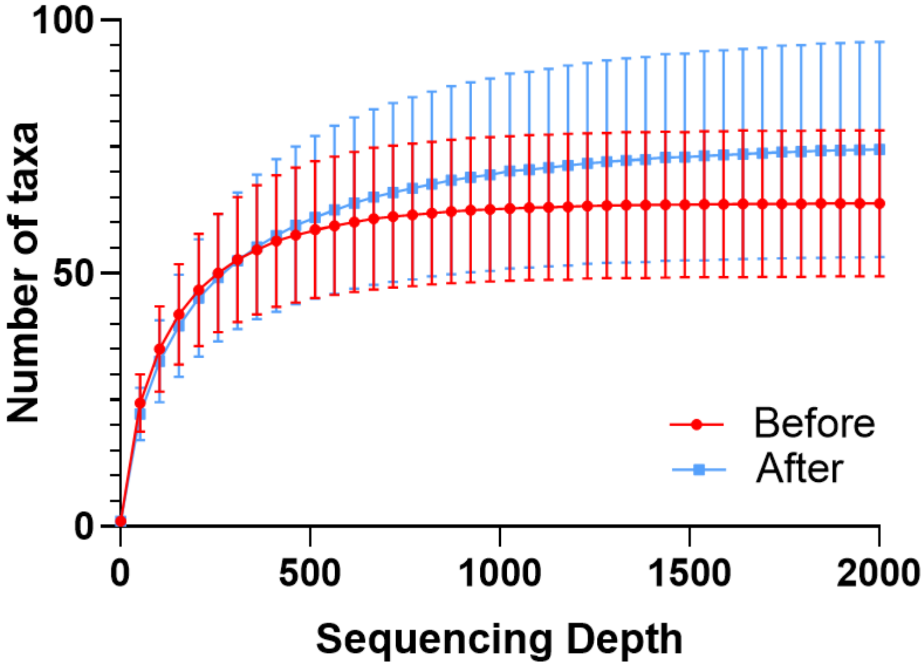

Supplementary Figure S3. Rarefaction analysis of gut microbiota in wild macaques classified based on 16S full-length sequencing using Oxford Nanopore Technologies.

The line graph showed the rarefaction curve representing the number of observed taxa over the sequencing depth identified in the macaque's gut microbiota before and after translocation.

Supplementary Table S3 The differential abundance species of gut microbiota before and after translocation into hygienic housing for 1 year analyzed by Linear discriminant analysis Effect Size (LEfSe) analysis LDA score > 4.5, *P*<0.05)

Species	*P* values	FDR	LDA Scores
Oscillibacter valericigenes	0.000866	0.002905	5.39
Dysgonomonas termitidis	4.87E-06	3.62E-05	5.29
Solitalea canadensis	3.62E-07	6.27E-06	5.16
Intestinimonas butyriciproducens	3.07E-05	0.000168	5.16
Eubacterium coprostanoligenes	1.18E-06	1.60E-05	5.03
Catabacter hongkongensis	2.96E-06	2.44E-05	4.98
Christensenella minuta	2.86E-05	0.000168	4.97
Ethanoligenens harbinense	1.23E-06	1.60E-05	4.89
Prevotella dentasini	0.003821	0.008832	4.84
Clostridium leptum	0.001271	0.003672	4.78
Eubacterium siraeum	0.006115	0.012231	4.70
Rikenella microfusus	2.33E-06	2.20E-05	4.69
Clostridium celatum	3.05E-06	2.44E-05	4.68
Clostridium thermosuccinogenes	0.010473	0.019109	4.63
Clostridium thermocellum	0.016867	0.028757	4.54
Anaerotruncus colihominis	0.013134	0.02355	4.51
Clostridium indolis	1.27E-07	2.64E-06	-4.53
Alloprevotella rava	0.000939	0.002959	-4.56
Lachnospira pectinoschiza	2.03E-06	2.20E-05	-4.58
Desulfotomaculum guttoideum	0.002962	0.007164	-4.65
Bifidobacterium pseudocatenulatum	0.002454	0.006076	-4.66
Blautia luti	0.001013	0.003099	-4.67
Brachyspira aalborgi	0.002344	0.005945	-4.68
Helicobacter cinaedi	0.001978	0.005143	-4.80
Prevotella stercorea	0.000337	0.001296	-4.83
Lactobacillus animalis	2.58E-08	8.95E-07	-4.90
Anaerovibrio lipolyticus	4.30E-08	1.12E-06	-4.95
Gemmiger formicilis	5.24E-05	0.000248	-5.03
Phascolarctobacterium succinatutens	2.07E-09	1.49E-07	-5.14
Helicobacter macacae	2.99E-05	0.000168	-5.25
Faecalibacterium prausnitzii	0.000925	0.002959	-5.40
Prevotella copri	8.70E-06	6.03E-05	-5.74
